# The relationship between weight-adjusted-waist index and total bone mineral density in adults aged 20-59

**DOI:** 10.3389/fendo.2023.1281396

**Published:** 2023-11-23

**Authors:** Meiqian Guo, Yi Lei, Xueqing Liu, Xiang Li, Yong Xu, Donghui Zheng

**Affiliations:** ^1^ Department of Nephrology, The Affiliated Huai’an Hospital of Xuzhou Medical University and Huai’an Second People’s Hospital, Huai’an, China; ^2^ Key Laboratory for Chronic Kidney Disease of Xuzhou Medical University, Xuzhou Medical University, Huai’an, China; ^3^ Huai'an Key Laboratory of Chronic Kidney Disease, The Affiliated Huai'an Hospital of Xuzhou Medical University and Huai'an Second People's Hospital, Huai’an, China; ^4^ Department of Clinical Laboratory, The Affiliated Huai’an Hospital of Xuzhou Medical University and Huai’an Second People’s Hospital, Huai’an, China

**Keywords:** weight-adjusted-waist index, NHANES, obesity, osteoporosis, total bone mineral density, adult

## Abstract

**Introduction:**

According to reports, obesity has a significant impact on bone health. And the weight-adjusted-waist index (WWI), superior to BMI and waist circumference (WC), is a new obesity indicator arising in recent years. This research investigated the relationship between WWI and total bone mineral density (BMD) for adults aged 20 to 59.

**Methods:**

Using data from the 2011–2018 NHANES, we looked into the independent link between WWI and total BMD as well as its nonlinearity using weighted multiple linear regression and smooth curve fitting. Two-stage linear regression models were employed to calculate the threshold effects. There were additional subgroup analyses and testing for interactions.

**Results:**

Multiple linear regression studies on a total of 10,372 individuals showed a significant inverse link between WWI and total BMD in adults between 20 and 59 [β = -0.04, 95% CI: (-0.04, -0.03), P<0.0001]. And smoking, race, and chronic kidney disease (CKD) had no significant effect on this negative connection (P for interaction >0.05). In addition, we found a nonlinear relationship between WWI and total BMD in diabetic and CKD patients, for which the saturation point was 11.38 cm/√kg in the CKD patient group and 10.29 cm/√kg in the diabetic patient group.

**Conclusion:**

Our analysis demonstrated a significant inverse association between WWI and total BMD in adults aged 20-59.

## 1 Introduction

A decrease in bone mineral density (BMD) and an increasing risk of bone fractures are two characteristics of the metabolic bone disease osteoporosis (1). It is described as a silent illness that precedes a fracture. Due to its severity, chronicity, and progression, osteoporosis has elevated as a public health problem (2–4). Worldwide, there are estimated to be more than 200 million cases of osteoporosis, and by 2025, it is expected that treating osteoporotic fractures will cost at least $25 billion (5). Therefore, it is essential to actively explore innovative methods of osteoporosis prevention as well as therapy to help safeguard public health.

In recent years, obesity has spread across the globe and is one of the main factors contributing to the rising prevalence of numerous chronic diseases (6–8). According to data, obesity incidence in America has climbed from 30.5% in 1999 to 41.9% in 2020, and the yearly medical expenditure associated with obesity is predicted to be at least $173 billion by 2025 (9). Although it was once widely accepted that obesity was positively correlated with either bone density or bone mass (10, 11), current research has revealed that the relationship might be the opposite (12, 13). Body mass index (BMI) is a typical measurement of obesity, although BMI only assesses overall obesity and ignores how body fat is distributed. Waist circumference (WC), like the BMI, is another commonly used indicator for evaluating obesity, but WC is more likely to indicate a condition associated with visceral fat and abdominal obesity (14–18). In 2018, Park et al. introduced a new measure of obesity called the weight-adjusted-waist index (WWI). WWI, which standardizes waist circumference for body weight by least-squares regression of log-transformed waist circumference on log-transformed body weight, has become a commonly used indicator for assessing central obesity in recent years (19). At the same time, studies have shown that WWI may be a sign of aging-related alterations in muscle and abdominal fat composition (20).

The purpose of our study was to determine the relationship between WWI and total BMD in US adults.

## 2 Materials and methods

### 2.1 Survey description and research population

To assess the physical and nutritional health of the American population, the National Center for Health Statistics (NCHS) conducts the National Health and Nutrition Examination Survey (NHANES), a nationwide cross-sectional study. Notably, the sample of NHANES is not a simple random sample. On the contrary, in order to collect health examination data of nationally representative non institutional US residents and civilians, NHANES adopted a complex stratified multi-stage sampling design (21). The sampling plan consisted of four stages: the primary sampling units (counties), a sample of area segments (blocks), dwelling units (households), and individuals. The data used in this study came from NHANES 2011 to 2018.

**Figure 1:**
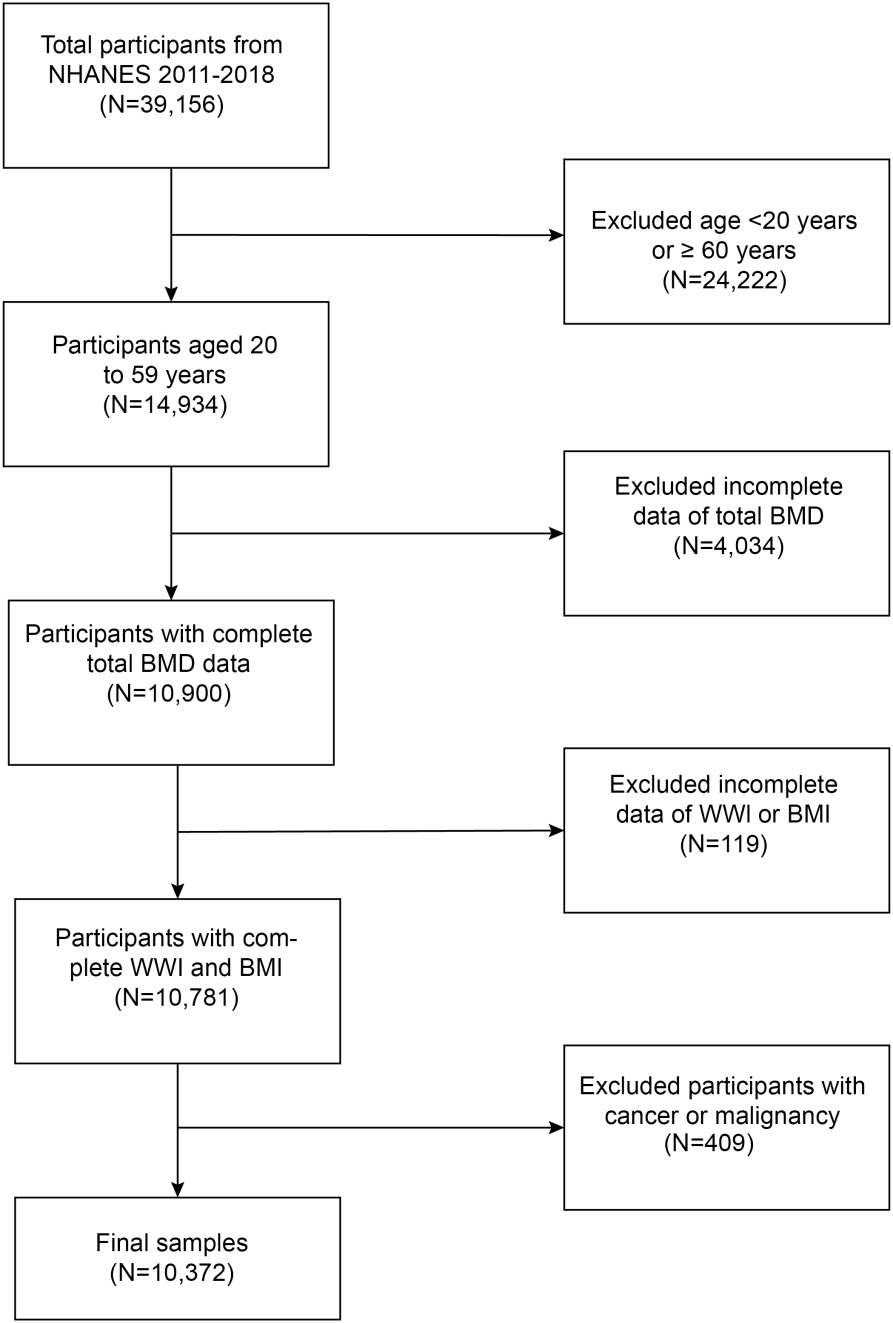
Flowchart of the sample selection.

Initially, a total of 39,156 individuals had been enlisted. And after removing those who were not between the ages of 20 and 59 years (n = 24,222), those with missing data on total bone mineral density (n = 4,034), those with missing data on WWI or BMI (n = 119), and those with a history of cancer or malignancy (n = 409), finally, 10,372 individuals participated in the study (**Figure 1**).

### 2.2 Research variables

WWI served as the exposure variable in this study. It is a new metric positively associated with excessive fat accumulation and computed by dividing WC by the square root of body weight. The WC was calculated in centimeters and the body weight was measured in kilograms. In this study, WWI was considered a continuous variable. In addition, we grouped subjects according to quartiles of WWI for further analysis. Total BMD, a continuous variable, was the outcome variable in this study. Total BMD is measured by qualified radiologists using dual-energy X-ray absorptiometry with professional equipment. According to the NHANES website, this organization utilizes strict implementation standards for every measurement. Taking BMD measurement as an example, not only does the organization retrain its technicians annually, but it also has dedicated staff on site to monitor the technicians’ specific operations. In addition, each technician’s performance code is tracked individually to minimize operational dependence of technicians to the greatest extent possible.

### 2.3 Selection of covariates

The covariates we looked at were age, race, sex, ratio of family income to poverty (PIR), education level, smoking status, BMI, fasting blood glucose, hemoglobin A1c, serum uric acid, serum urea nitrogen, vitamin D (25OHD2 + 25OHD3), total calcium, phosphorus, serum creatinine, serum albumin, total cholesterol, triglycerides, low-density lipoprotein cholesterol (LDL-C), high-density lipoprotein cholesterol (HDL-C), alanine aminotransferase (ALT), aspartate aminotransferase (AST), albumin creatinine ratio (ACR) and estimated glomerular filtration rate [eGFR, ml/min/1.73m^2^, calculated from the Chronic Kidney Disease Epidemiology Collaboration (CKD-EPI Cr) equation (22)]. Chronic kidney disease (yes/no) and diabetes (yes/no) health status disparities are also mentioned. Albuminuria was defined as ACR >30 mg/g, low eGFR was defined as eGFR <60 ml/min/1.73 m^2^, and albuminuria or a low eGFR were used to diagnose chronic kidney disease (CKD). Diabetes mellitus has been defined as using hypoglycemic medication or having ever received a diagnosis of the condition from a doctor, fasting glucose ≥ 126 mg/dl, or plasma glucose ≥ 200 mg/dl at 2 hours postprandial, hemoglobin A1c level ≥ 6.5%. The BMI ranges for normal weight, overweight, and obesity are <25, 25-29.9, and ≥30 kg/m^2^, respectively. The official website of the NHANES (https://www.cdc.gov/nchs/nhanes/) provides information on how to interpret, measure, and calculate each variable.

### 2.4 Statistic analysis

Categorical variables in this study were expressed as percentages and differences between groups were assessed using the weighted chi-square test. Continuous variables were expressed as mean ± standard deviation and differences between groups were assessed using weighted Student’s t-test. By using weighted multiple linear regression analysis, we assessed a linear link between WWI and BMD in the subjects in three models. This method took into account the different variances of different observations, gave them different weights, and provided more reliable predictions than ordinary linear regression. Model 1 required no adjustment for variables. Model 2 incorporated three common demographic variables, including race, sex, and age. Model 3 adjusted for all covariates listed in **Table 1** except for waist circumference and weight, which included the three demographic variables addressed in Model 2, as well as a number of variables that have been considered to be associated with obesity or BMD in previous studies (23–27). We have performed a sensitivity analysis of each model, assessed the goodness of fit, and investigated the models for the presence of collinearity. The results showed that the results of each model were stable, with good fit and no obvious collinearity issues. In addition, subgroup analyses were performed in this study. Subsequently, to explore the nonlinear association between WWI and total BMD, we used generalized additivity modeling (GAM) and smoothed curve fitting (28). Smoothing curves and GAMs are common techniques used to capture nonlinear relationships between predictor and response variables and can help identify key trends and patterns in the data. We also used this method to probe whether nonlinear associations also existed in each stratified subgroup. If nonlinear associations were found, two-stage regression analysis was used to probe for inflection points to further elucidate the correlation between WWI and total BMD (29). Two stage regression is a nonlinear regression technique aimed at fitting the sudden changes in the response of the dependent variable to the explanatory variable at inflection points (also known as breakpoints or thresholds) in nonlinear relationships, which helps to capture the changes in data at different stages. The appearance of a turning point suggests that there may be some potential mechanism near this point that leads to a change in the influence of the independent variable on the dependent variable. Statistical evaluations in this survey were performed using R version 4.2.3 (http://www.r-project.org) and EmpowerStats (http://www.empowerstats.com), and p<0.05 was regarded as statistically significant.

**Table 1:** Basic characteristics of participants by weight-adjusted-waist index quartile.

Characteristics	Q1 (8.37 - 10.27)	Q2 (10.28 - 10.8)	Q3 (10.81 - 11.35)	Q4 (11.36 - 14.04)	*P-value*
	N= 2,582	N= 2,548	N= 2,620	N= 2,622	
Age (year)	32.72 ± 10.44	38.75 ± 11.09	41.55 ± 11.06	43.50 ± 11.08	<0.0001
Sex (%)					<0.0001
Male	61.82	55.71	51.16	34.63	
Female	38.18	44.29	48.84	65.37	
Race (%)					<0.0001
Mexican American	5.15	9.89	12.66	16.01	
Other Hispanic	5.87	7.44	8.97	8.38	
Non-Hispanic White	62.78	62.35	57.36	57.54	
Non-Hispanic Black	16.06	9.41	10.07	10.52	
Other Race	10.14	10.91	10.93	7.54	
Education level(%)					<0.0001
Less than high school	1.77	3.10	5.03	6.77	
High school or GED	25.37	28.20	34.32	37.66	
Above high school	72.86	68.68	60.61	55.56	
Unknown	0.00	0.01	0.03	0.00	
Smoking status(%)					<0.0001
Never	64.28	60.50	57.51	55.46	
Former	15.14	18.90	21.00	21.29	
Current	20.54	20.57	21.45	23.23	
Unknown	0.04	0.03	0.04	0.02	
Diabetes (%)					<0.0001
Yes	2.14	5.51	10.37	20.18	
No	97.86	94.49	89.63	79.82	
CKD (%)					<0.0001
Yes	6.11	6.97	10.25	13.88	
No	93.89	93.03	89.75	86.12	
PIR	3.02 ± 1.68	3.08 ± 1.65	2.94 ± 1.67	2.65 ± 1.66	<0.0001
Albumin (g/dl)	4.45 ± 0.34	4.36 ± 0.32	4.29 ± 0.32	4.17 ± 0.32	<0.0001
ALT (U/L)	22.26 ± 14.74	25.63 ± 18.30	28.42 ± 23.61	28.30 ± 20.30	<0.0001
AST (U/L)	24.44 ± 13.64	24.47 ± 11.69	26.27 ± 23.46	25.62 ± 19.12	0.0002
Vitamin D (nmol/L)	69.25 ± 26.38	67.49 ± 26.09	65.44 ± 25.66	62.20 ± 25.15	<0.0001
Total calcium (mg/dl)	9.43 ± 0.32	9.38 ± 0.33	9.35 ± 0.36	9.32 ± 0.36	<0.0001
Phosphorus (mg/dl)	3.79 ± 0.56	3.71 ± 0.56	3.70 ± 0.55	3.67 ± 0.56	<0.0001
Serum glucose (mg/dl)	88.41 ± 16.62	94.21 ± 28.87	99.01 ± 32.76	107.50 ± 42.93	<0.0001
Glycohemoglobin (%)	5.25 ± 0.50	5.41 ± 0.79	5.58 ± 0.93	5.87 ± 1.20	<0.0001
Uric acid (mg/dl)	5.16 ± 1.26	5.33 ± 1.39	5.38 ± 1.37	5.47 ± 1.41	<0.0001
BUN(mg/dl)	12.79 ± 4.06	12.99 ± 4.24	12.71 ± 4.28	12.51 ± 4.39	0.0012
Creatinine (mg/dl)	0.90 ± 0.25	0.87 ± 0.31	0.84 ± 0.31	0.79 ± 0.28	<0.0001
ACR (mg/g)	9.91 ± 18.80	10.09 ± 19.00	12.88 ± 29.42	16.57 ± 34.19	<0.0001
eGFR (mL/min/1.73 m2)	94.03 ± 18.95	92.96 ± 20.42	94.21 ± 21.58	96.48 ± 23.99	<0.0001
Triglyceride (mg/dl)	89.03 ± 68.80	115.25 ± 109.55	132.79 ± 121.90	143.67 ± 146.75	<0.0001
Total cholesterol (mg/dl)	177.89 ± 34.37	191.64 ± 37.18	197.74 ± 41.84	197.04 ± 43.39	<0.0001
LDL-C (mg/dl)	102.97 ± 30.47	113.57 ± 32.63	120.48 ± 36.79	119.44 ± 33.31	<0.0001
Direct HDL-C (mg/dl)	57.42 ± 16.14	53.93 ± 15.60	50.11 ± 14.83	49.05 ± 14.24	<0.0001
Weight (kg)	72.86 ± 15.03	79.42 ± 17.94	84.84 ± 19.33	93.89 ± 24.09	<0.0001
Waist circumference (cm)	83.51 ± 9.32	93.46 ± 10.74	101.27 ± 11.69	113.85 ± 15.54	<0.0001
BMI (kg/m^2^)	24.29 ± 4.20	27.24 ± 4.90	29.76 ± 5.50	34.46 ± 7.29	<0.0001
Total BMD (g/cm^2^)	1.14 ± 0.11	1.12 ± 0.10	1.11 ± 0.11	1.09 ± 0.10	<0.0001

Mean ± SD for continuous variables: the p-value was calculated by the weighted linear regression model. (%) for categorical variables: the p-value was calculated by the weighted chi-square test.

Q, quartile; GED, general educational development; CKD, chronic kidney disease; PIR, ratio of family income to poverty; BMI, body mass index; LDL-C, low-density lipoprotein cholesterol; BMD, bone mineral density; HDL-C, direct high-density lipoprotein cholesterol; AST, aspartate transaminase; ALT, alanine transaminase; Vitamin D, 25OHD2 + 25OHD3;BUN, blood urea nitrogen; ACR, albumin: creatinine ratio; eGFR, estimated-glomerular filtration rate.

## 3 Results

### 3.1 Baseline characteristics

There were 10,372 participants in this research, and their average age was 38.98 ± 11.65. 51.25% of them were men, and 48.75% were women. WWI average (SD) was 10.79 ± 0.78 cm/√kg, while the total BMD average (SD) was 1.12 ± 0.11 g/cm2.The interquartile ranges of WWI were 8.37 - 10.27, 10.28 - 10.80, 10.81 - 11.35, 11.36 - 14.04. As illustrated in **Table 1**, our data demonstrated that individuals in the highest WWI quartiles were significantly more inclined to be elder, female, and smokers. They also showed a greater risk of diabetes and CKD and had higher levels of ALT, AST, uric acid, glycohemoglobin, serum glucose, ACR, eGFR, triglyceride, total cholesterol, and LDL-C as compared to individuals in other categories. On the contrary, they had lower levels of education, PIR, albumin, BUN, blood creatinine, direct HDL-C, 25OHD2 + 25OHD3, total calcium, blood phosphorus, and total BMD (p<0.05). The baseline characteristics of subjects according to WWI quartiles are shown in **Table 1**.

### 3.2 Total BMD has a negative connection with WWI

Multiple regression analysis found that total BMD has a negative connection with WWI. The association was notable in both Model 1 (β= -0.03; 95% CI: -0.03,-0.03; p<0.0001) and Model 2 (β= -0.01; 95% CI: -0.02,-0.01, p<0.0001). In Model 1, every increase in WWI by one unit was closely related to a decrease of 0.03 units in total BMD, while in Model 2, this effect value was -0.01. And in model 3, the fully adjusted model, this negative correlation persisted, suggesting that a one-unit increase in WWI was strongly associated with a 0.04-unit decrease in total BMD(β= -0.04;95% CI: -0.04,-0.03, p<0.0001). After further converting WWI from continuous to categorical (quartiles), the total BMD of subjects in the upper quartile of WWI was 0.06 g/cm^2^ lower than that of subjects in the bottom quartile in Model 1 (β=-0.06; 95% CI: -0.07, -0.06; p<0.0001), whereas in Models 2 and 3, the values for this effect were -0.03 (β=-0.03; 95% CI: -0.03, -0.02; p<0.0001) and -0.07 (β=-0.07; 95% CI: -0.08, -0.06; p<0.0001), respectively, and all these results were statistically significant (all p for trend <0.0001) (**Table 2**).

**Table 2:** Association between weight-adjusted-waist index (cm/√kg) and total bone mineral density (g/cm^2^).

Exposure	Model 1 [β (95% CI)], *P* value	Model 2 [β (95% CI)], *P* value	Model 3 [β (95% CI)], *P* value
WWI (continuous)	-0.03 (-0.03, -0.03), <0.0001	-0.01 (-0.02, -0.01),<0.0001	-0.04 (-0.04, -0.03),<0.0001
WWI (quartile)			
Quartile 1	Reference	Reference	Reference
Quartile 2	-0.03 (-0.03, -0.02),<0.0001	-0.01 (-0.02, -0.01),0.0001	-0.02 (-0.03, -0.01), <0.0001
Quartile 3	-0.04 (-0.05, -0.04),<0.0001	-0.02 (-0.02, -0.01),<0.0001	-0.04 (-0.05, -0.03),<0.0001
Quartile 4	-0.06 (-0.07, -0.06),<0.0001	-0.03 (-0.03, -0.02),<0.0001	-0.07 (-0.08, -0.06),<0.0001
*p* for trend	<0.0001	<0.0001	<0.0001

Model 1: no covariates were adjusted.

Model 2: age, sex, and race were adjusted.

Model 3: age, sex, race, education, smoking status, diabetes, CKD, PIR, BMI, albumin, ALT, AST, vitamin D, BUN, total calcium, creatinine, serum glucose, glycohemoglobin, phosphorus, uric acid, eGFR, ACR, total cholesterol, triglyceride, LDL-C, and direct HDL-C were adjusted.

CKD, chronic kidney disease; PIR, ratio of family income to poverty; LDL-C, low-density lipoproteincholesterol; HDL-C, high-density lipoprotein cholesterol; BMI, body mass index, AST, aspartate aminotransferase; ALT, alanine aminotransferase; ACR, albumin:creatinine ratio; eGFR, estimated-glomerular filtration rate; BUN, blood urea nitrogen; vitamin D, 25OHD2 + 25OHD3.

### 3.3 Subgroup analysis

As shown in **Table 3**, in subgroup analyses stratified by age, sex, race, BMI, smoking status, diabetes mellitus, and CKD status, we found that the negative association between WWI and BMD persisted in all subgroups. In addition, stratified analyses found stronger associations between increased WWI and decreased total BMD in elder, male, overweight, and non-diabetic patients (P for interaction <0.05). For example, in the stratified by diabetes status subgroup analyses, the absolute effect size was significantly larger in nondiabetic patients than in diabetic subjects. This meant that in non-diabetic subjects, each unit increase in WWI was strongly associated with a 0.04 unit decrease in total BMD (β=-0.04; 95% CI: -0.05,-0.04, P<0.0001), whereas in diabetic patients, it was associated with only a 0.02 unit decrease in total BMD (β=-0.02; 95% CI: - 0.04,- 0.01, P=0.0026). In contrast, race, smoking status, and CKD status did not significantly alter this negative correlation(p for interaction >0.05).

**Table 3:** Subgroups analyses of the association between weight-adjusted-waist index (cm/√kg) and total bone mineral density (g/cm^2^).

	β (95% CI), *p* for trend	*p* for interaction
Stratified by age		0.0093
20-39 years old	-0.04 (-0.04, -0.03), <0.0001	
40-59 years old	-0.05 (-0.05, -0.04), <0.0001	
Stratified by sex		<0.0001
Male	-0.07 (-0.07, -0.06), <0.0001	
Female	-0.03 (-0.04, -0.02), <0.0001	
Stratified by race		0.1535
Mexican American	-0.02 (-0.04, -0.01), 0.0016	
Other Hispanic	-0.04 (-0.05, -0.02), 0.0002	
Non-Hispanic White	-0.04 (-0.05, -0.04), <0.0001	
Non-Hispanic Black	-0.04 (-0.05, -0.02), <0.0001	
Other Race	-0.04 (-0.06, -0.03), 0.0009	
Stratified by BMI		0.0005
Normal weight	-0.04 (-0.04, -0.03), <0.0001	
Overweight	-0.05 (-0.06, -0.04), <0.0001	
Obese	-0.03 (-0.04, -0.02), <0.0001	
Stratified by smoking status		0.9419
Never	-0.04 (-0.05, -0.04), <0.0001	
Former	-0.04 (-0.05, -0.03), <0.0001	
Current	-0.04 (-0.05, -0.03), <0.0001	
Diabetes		0.0102
Yes	-0.02 (-0.04, -0.01), 0.0026	
No	-0.04 (-0.05, -0.04), <0.0001	
CKD		0.0546
Yes	-0.03 (-0.04, -0.02), <0.0001	
No	-0.04 (-0.05, -0.04), <0.0001	

Subgroup analyses including age, sex, race, BMI, smoking status, diabetes status and CKD status. The model adjusted for covariates such as age, sex, race, education, diabetes, CKD, smoking status, PIR, albumin, ALT, AST, vitamin D, BUN, total calcium, creatinine, serum glucose, phosphorus, uric acid, ACR, eGFR, glycohemoglobin, total cholesterol, triglycerides, LDL-C, and direct HDL-C, but the model did not adjust for the stratification variables themselves.

### 3.4 Non-linearity and saturation effect analysis

**Figure 2:**
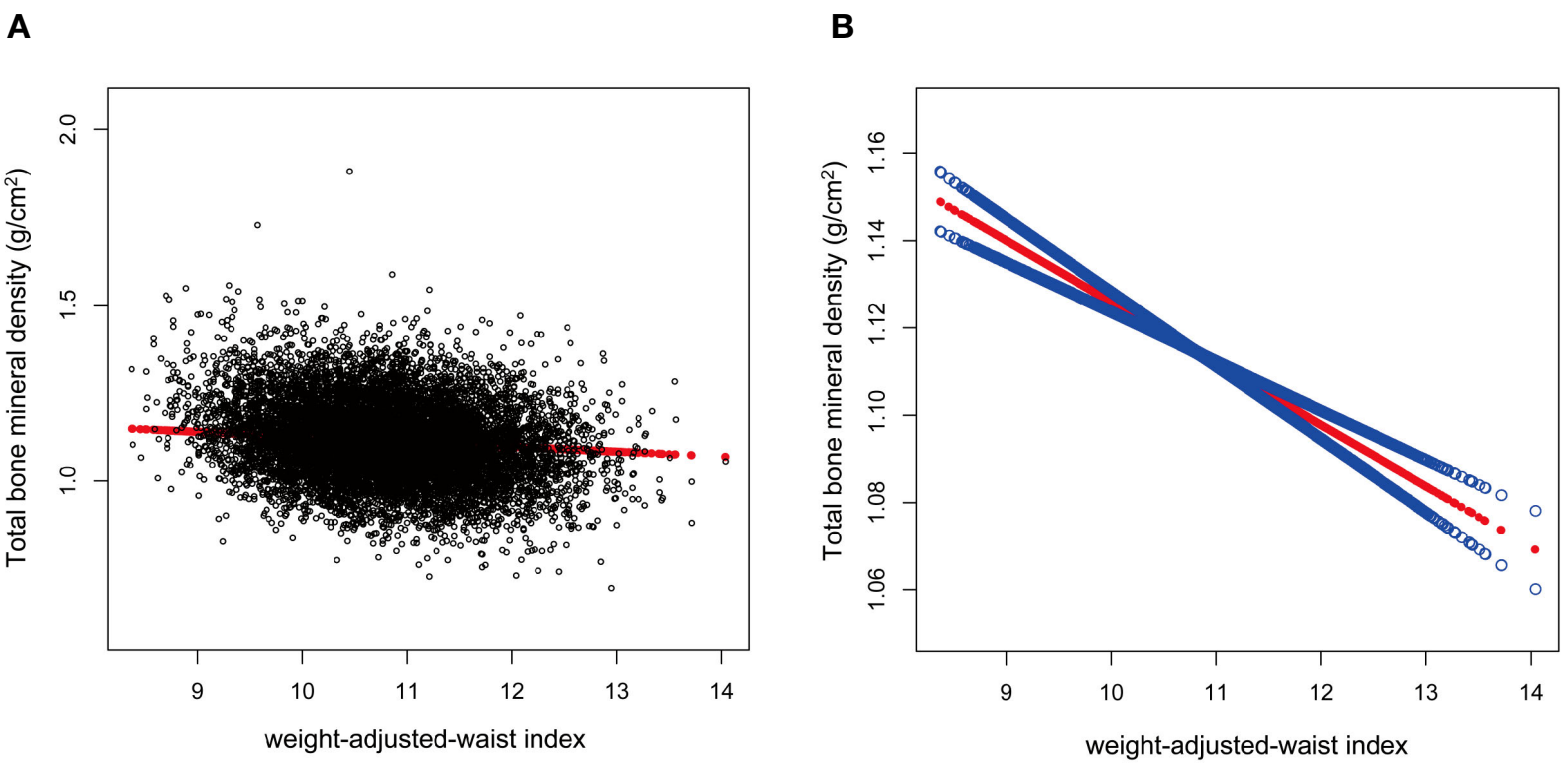
Association between weight-adjusted-waist index (cm/√kg) and total bone mineral density (g/cm^2^). The association between WWI and total BMD. **(A)** Each black point represents a sample. **(B)** The solid red line represents the smooth curve fit between variables. Blue bands represent the 95% of confidence interval from the fit.WWI, weight-adjusted-waist index; total BMD, total bone mineral density.

**Figure 3:**
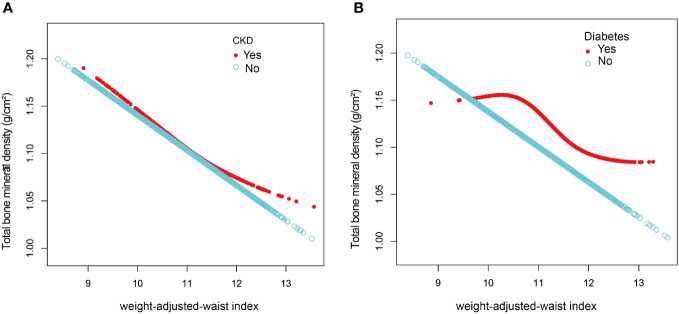
The association between weight-adjusted-waist index and total bone mineral density stratified by CKD **(A)** and diabetes **(B)**. The model adjusted for covariates such as age, sex, race, education, diabetes, CKD, smoking status, PIR, albumin, ALT, AST, vitamin D, BUN, total calcium, creatinine, serum glucose, phosphorus, uric acid, ACR, eGFR, glycohemoglobin, total cholesterol, triglycerides, LDL-C, and direct HDL-C, but the model did not adjust for the stratification variables themselves.

After the generalized model smoothing curve fitting, the non-linear relationship between WWI and total BMD was shown in **Figure 2**. Subsequently, the nonlinear relationships in each stratum were further examined using GAM and smoothed curve fitting, and we found nonlinear associations between WWI and BMD in the diabetic and CKD populations (**Figure 3**). By threshold effect analysis, we calculated an inflection point (K) of 11.38 cm/√kg for the CKD population. To the left of the inflection point, WWI was negatively correlated with total BMD (β = -0.06, 95% CI: -0.08, -0.03), and no statistically significant relationship was observed to the right of the inflection point (β = 0.01, 95% CI: -0.02, 0.04). In other words, in population with CKD, an increase in WWI was significantly correlated with a decrease in total BMD until WWI reached 11.38 cm/√kg. However, after WWI reached 11.38 cm/√kg, there was no significant correlation between WWI and BMD in CKD patients. In the diabetic population, this inflection point was 10.29 cm/√kg. When WWI was less than 10.29 cm/√kg, there was no significant correlation between WWI and total BMD in diabetic patients. However, after WWI reached 10.29 cm/√kg, each increase in WWI in diabetic patients was significantly associated with a 0.03 unit decrease in their total BMD (**Table 4**).

**Table 4:** Threshold effect analysis of weight-adjusted-waist index on total bone mineral density in individuals with CKD and diabetes by two-stage linear regression model.

Total bone mineral density	Adjusted β (95% CI),P value
CKD	
Fitting by the standard linear model	-0.03 (-0.05, -0.02), <0.0001
Fitting by the two-piecewise linear model	
Inflection point	11.38
WWI<11.38	-0.06 (-0.08, -0.03), <0.0001
WWI>11.38	0.01 (-0.02, 0.04), 0.6894
Log likelihood ratio	0.001
Diabetes	
Fitting by the standard linear model	-0.02 (-0.04, -0.00), 0.0196
Fitting by the two-piecewise linear model	
Inflection point	10.29
WWI<10.29	0.08 (-0.02, 0.18), 0.1300
WWI>10.29	-0.03 (-0.05, -0.01), 0.0041
Log likelihood ratio	0.043

The model adjusted for covariates such as age, sex, race, education, diabetes, CKD, smoking status, PIR, albumin, ALT, AST, vitamin D, BUN, total calcium, creatinine, serum glucose, phosphorus, uric acid, ACR, eGFR, glycohemoglobin, total cholesterol, triglycerides, LDL-C, and direct HDL-C, but the model did not adjust for the stratification variables themselves.

## 4 Discussion

We examined the connection between WWI and total BMD in U.S. adults aged 20 to 59 years in this cross-sectional research of 10,372 subjects. Higher WWI was shown to be substantially linked with lower total BMD. This negative correlation was found in all subgroups, according to subgroup analyses and interaction tests, whereas smoking, race, and CKD had no significant effect on this negative connection. Notably, we discovered a non-linear association between WWI and total BMD in the populations of people with diabetes and CKD, and we further computed an inflection point (K) of 11.38 cm/√kg in the CKD group and 10.29 cm/√kg in the population of those with diabetes. This suggested that in populations with CKD and diabetes, there may be some underlying mechanism influencing the role of WWI on total BMD near the corresponding inflection point.

Obesity and overweight have previously been identified as preventative factors for fractures and osteoporosis. BMI and WC are two internationally accepted parameters for identifying obesity, and numerous earlier studies have found positive connections between BMI, WC, and BMD (11, 30). After adjusting for other factors, researchers discovered that BMI was positively linked with lumbar spine BMD in a study involving 2,218 adults between the ages of 40 and 59 (10). In a study involving 2,903 individuals aged 50 and above, researchers found a positive correlation between BMI and WC and femoral neck BMD, with BMI saturation values of 24.3 kg/m^2^ (31). The link between fat and osteoporosis is complicated, though, and contradictory results are frequently reported. In their study of 5,801 adults over the age of 60, Chen et al. concluded that abdominal obesity was a risk cause for poor bone health in older adults, regardless of BMI, and that the relationship between WC and femoral neck BMD had an inverted U-shaped pattern having a 95 cm inflection point at WC (32). In all age subgroups with normal and overweight BMI, WC was significantly and negatively associated with BMD at all sites in men according to Yin et al. In contrast, in low BMI subjects, WC did not affect BMD in any age group or site (33). A growing number of experts believe that BMI and WC are insufficient as accurate measurements of obesity since they cannot differentiate between lean body mass and fat mass and are subject to age, sex, and racial differences (34).

In 2018, Park et al. suggested WWI as a new obesity metric, which standardizes WC for body weight to make it easier to assess (19). WWI is extensively employed in a multiracial setting since it has little interracial variation and has been found to reflect changes in abdominal composition with age, such as an increase in abdominal fat content and a reduction in muscle mass (20). The index has since been studied in a variety of fields, including obesity and cardiovascular disease (19, 35–39). The association between WWI and BMD or osteoporosis in various age groups has been documented in earlier studies. Wang et al. came up with an inverse correlation between teenage WWI and total BMD using data from the 2011-2018 NHANES on 6,923 adolescents (aged 8 to 19) (26). With the exception of age, this negative association remained constant across all subgroups, including sex, race, and diabetes status. In an investigation involving participants between the ages of 65 and 80, Lin et al. discovered a positive connection between WWI and osteoporosis in older persons (40). Our analysis discovered a significant inverse relationship between total BMD and WWI among individuals aged 20 to 59 years, which is consistent with earlier findings.

In our study, we also carried out subgroup analyses. According to our findings, non-diabetic patients with greater WWI levels had a greater chance of acquiring osteoporosis. This is consistent with the findings of various earlier investigations. In the US, a study involving 9,661 individuals discovered that individuals with impaired glucose metabolism had higher BMD (24). Furthermore, diabetes patients had significantly greater femur BMD levels than controls, according to a retrospective study with 751 female participants (41). According to the results above, diabetes status may have a positive effect on BMD. Contrarily, stratified studies revealed that the presence or absence of CKD has little impact on the association between WWI and BMD. Previous research has demonstrated that osteoporosis prevalence is much higher in sufferers of CKD than in age-matched controls, with a loss in BMD launching at an early stage and CKD as a separate risk factor for osteoporosis (42–44). The complex and varied mechanisms that underlie this phenomenon are typically attributed to abnormalities in the metabolism of calcium and phosphorus, secondary hyperparathyroidism, abnormalities in bone conversion, deficiencies in 1,α-hydroxylase and vitamin D, metabolic acidosis, uremic toxins, microinflammatory states, among others (45–47). It might seem contrary to what we discovered. Based on a study from China, eGFR was negatively linked with osteoporosis in stage 3-5 CKD patients (48). Another research demonstrated that when renal function declines, CKD patients lose BMD, and their odds of fracture increase (49). According to this, different stages of CKD may have various outcomes, despite either the presence or absence of CKD has little influence on the link between WWI and BMD (50, 51).

Numerous studies have been conducted in recent years on the relationship between obesity and osteoporosis. The probable molecular mechanisms underpinning how obesity affects bone health, nevertheless, are still not fully known. The potential mechanisms may comprise part of the following. First, adipose tissue from obese individuals releases cytokines such as IL-1B, TNF-α, and IL-6, which alone or in combination can activate intracellular signaling pathways that could bring about bone loss (52). Secondly, studies have demonstrated that imbalances in the control of the gut microbiota are related to obesity (53). Adipose tissue produces more pro-inflammatory cytokines when an individual is fat, causing increased gut barrier permeability and raised serum levels of the endotoxin lipopolysaccharide (LPS). LPS is an effective inducer of bone deterioration, based on studies (54). Leptin and adiponectin are the two major adipokines generated by adipose tissue. Obesity may induce adipose tissue to release more leptin and less adiponectin, and researchers have demonstrated that this can enhance the activity of osteoclasts and cause bone loss (55, 56). Animal studies demonstrated that obesity brought on by a high-fat diet could cause femoral trabecular and cortical bone loss in male rats. This is most likely because obesity leads to oxidative stress in the body, which in turn leads to iron death in osteoprogenitor cells and endothelial cells (57). Furthermore, there is mounting proof that obesity might change the bone marrow microenvironment and thus impair certain properties of bone marrow-derived mesenchymal stem cells (BMSCs), such as the ability of BMSCs to produce bone (58, 59).

Our study has certain strengths. First, the majority of studies have concentrated on the bone health of postmenopausal women and older populations. And there is limited information on the association between WWI and BMD in young adults. Our study selected a national population aged 20 to 59 to shed some light on this aspect. Second, we chose a comprehensive national sample, so the sample size was large enough to allow for subgroup analysis of diabetes and CKD populations. But this research contains several drawbacks. The cross-sectional design of our analysis restricts the conclusion that WWI and adult total BMD are causally related. Then, we did not consider whether different calendar period years would have some impact on this relationship. We look forward to future research that can fill this gap. In addition, despite the sophisticated sampling methodology used in this study, it may still not fully reflect that the true situation exists, and therefore larger studies are needed in the future to test our findings. Beyond the covariates considered in this paper, there are many other factors that have been shown to influence bone health, so more comprehensive studies are needed in the future to validate our findings.

## 5 Conclusion

Our study found a significant negative correlation between WWI and total BMD in adults aged 20 to 59 years, whereas smoking, race, and CKD had no significant effect on this negative connection. More study is required to validate our position.

## Data availability statement

Publicly available datasets were analyzed in this study. This data can be found here: https://www.cdc.gov/nchs/nhanes/index.htm.

## Ethics statement

The studies involving humans were approved by the National Center for Health Statistics. The studies were conducted in accordance with the local legislation and institutional requirements. The participants provided their written informed consent to participate in this study. Written informed consent was obtained from the individual(s) for the publication of any potentially identifiable images or data included in this article.

## Author contributions

MG: Conceptualization, Writing – original draft, Data curation. YL: Investigation, Methodology, Writing – original draft. XQL: Investigation, Methodology, Writing – original draft. XL: Writing – review and editing. YX: Writing – review and editing. DZ: Conceptualization, Writing – review and editing.
