# Supplementary material for: The relationship between weight-adjusted-waist index and total bone mineral density in adults aged 20-59
Source: Front Endocrinol (Lausanne). 2023 Nov 23;14:1281396. doi: 10.3389/fendo.2023.1281396 (PMC10701523; doi:10.3389/fendo.2023.1281396)
Supplement: Supplementary file 1 [file Table_1.docx]

| **Variables** | **Model 1** | **Model 2** | **Model 3** |
| --- | --- | --- | --- |
|  | **β (95% CI), P value** | **β (95% CI), P value** | **β (95% CI), P value** |
| Age (year) | -0.00 (-0.00, -0.00), <0.0001 | -0.00 (-0.00, -0.00), <0.0001 | -0.00(-0.00, -0.00), <0.0001 |
| Female (versus male) | -0.07 (-0.07, -0.06), <0.0001 | -0.06 (-0.07, -0.06), <0.0001 | -0.08(-0.09,-0.07), <0.0001 |
| race (versus Mexican American) |  |  |  |
| Other Hispanic | 0.00 (-0.01, 0.01), 0.8845 | 0.00 (-0.00, 0.01), 0.3775 | -0.00 (-0.02, 0.01), 0.4443 |
| Non-Hispanic White | 0.02 (0.02, 0.03), <0.0001 | 0.02 (0.02, 0.03), <0.0001 | 0.01 (-0.00, 0.02), 0.0884 |
| Non-Hispanic Black | 0.08 (0.08, 0.09), <0.0001 | 0.08 (0.08, 0.09), <0.0001 | 0.08 (0.07, 0.09), <0.0001 |
| Other Races | -0.00 (-0.01, 0.00), 0.4456 | -0.00 (-0.01, 0.00), 0.1597 | -0.01 (-0.02, 0.00), 0.1777 |
| Education level (versus less than high school) |  |  |  |
| High school or GED | 0.04 (0.03, 0.04), <0.0001 | 0.01 (0.00, 0.02), 0.0100 | 0.00 (-0.01, 0.01), 0.9861 |
| Above high school | 0.04 (0.03, 0.04), <0.0001 | 0.02 (0.01, 0.03), <0.0001 | 0.00 (-0.01, 0.01), 0.9309 |
| Others | -0.02 (-0.17, 0.13), 0.8187 | -0.02 (-0.15, 0.12), 0.8173 | 0 |
| Smoke (versus never) |  |  |  |
| Former | 0.00 (-0.00, 0.01), 0.3341 | -0.00 (-0.01, 0.00), 0.7011 | -0.00 (-0.01, 0.00), 0.4389 |
| Current | 0.01 (0.00, 0.02), 0.0002 | -0.00 (-0.01, 0.00), 0.0545 | 0.00 (-0.01, 0.01), 0.8288 |
| Unknown | -0.01 (-0.09, 0.08), 0.8966 | -0.04 (-0.12, 0.03), 0.2392 | -0.09 (-0.22, 0.04), 0.1756 |
| Diabetes (no versus yes) | -0.00 (-0.01, 0.00), 0.1480 | -0.01 (-0.02, -0.01), <0.0001 | 0.00 (-0.01, 0.02), 0.7105 |
| CKD(no versus yes) | 0.01 (-0.00, 0.01), 0.1348 | -0.00 (-0.01, 0.01), 0.6581 | 0.00 (-0.01, 0.02), 0.4401 |
| BMI (kg/m^2^) | 0.00 (0.00, 0.00), <0.0001 | 0.00 (0.00, 0.00), <0.0001 | 0.00 (0.00, 0.00), <0.0001 |
| PIR | 0.00 (0.00, 0.00), 0.0012 | 0.00 (0.00, 0.01), <0.0001 | 0.00 (0.00, 0.01), 0.0012 |
| Albumin (g/dl) | 0.01 (0.00, 0.02), 0.0070 | -0.02 (-0.03, -0.01), <0.0001 | -0.01 (-0.02, 0.01), 0.3496 |
| ALT (U/L) | 0.00 (0.00, 0.00), 0.0020 | -0.00 (-0.00, 0.00), 0.1715 | -0.00 (-0.00, 0.00), 0.1300 |
| AST (U/L) | 0.00 (-0.00, 0.00), 0.0664 | -0.00 (-0.00, 0.00), 0.2885 | 0.00 (-0.00, 0.00), 0.5636 |
| Vitamin D (nmol/L) | -0.00 (-0.00, -0.00), 0.0003 | 0.00 (0.00, 0.00), <0.0001 | 0.00 (0.00, 0.00), <0.0001 |
| Total calcium (mg/dl) | 0.01 (0.00, 0.02)，0.0029 | -0.01 (-0.02, -0.01), <0.0001 | -0.01 (-0.02, -0.00), 0.0265 |
| Phosphorus (mg/dl) | -0.00 (-0.01, 0.00), 0.1202 | -0.00 (-0.00, 0.00), 0.8134 | -0.00 (-0.01, 0.00), 0.1779 |
| Serum glucose (mg/dl) | 0.00 (0.00, 0.00), 0.0082 | 0.00 (0.00, 0.00), <0.0001 | 0.00 (0.00, 0.00), 0.0117 |
| Glycohemoglobin (%) | 0.00 (0.00, 0.01), 0.0004 | 0.00 (0.00, 0.01), <0.0001 | -0.00 (-0.01, 0.00), 0.6248 |
| Uric acid (mg/dl) | 0.02 (0.01, 0.02), <0.0001 | 0.00 (0.00, 0.01), <0.0001 | -0.00 (-0.00, 0.00), 0.6455 |
| BUN(mg/dl) | 0.00 (0.00, 0.00), <0.0001 | 0.00 (0.00, 0.00), <0.0001 | 0.00 (-0.00, 0.00), 0.4401 |
| Creatinine (mg/dl) | 0.05 (0.05, 0.06), <0.0001 | 0.01 (0.01, 0.02), 0.0001 | -0.02 (-0.04, 0.01), 0.1942 |
| ACR (mg/g) | 0.00 (-0.00, 0.00), 0.3963 | 0.00 (-0.00, 0.00), 0.8708 | -0.00 (-0.00, 0.00), 0.5766 |
| eGFR (mL/min/1.73 m^2^) | -0.00 (-0.00, -0.00), <0.0001 | -0.00 (-0.00, -0.00), <0.0001 | -0.00 (-0.00, -0.00), <0.0001 |
| Triglyceride (mg/dl) | -0.00 (-0.00, 0.00), 0.9524 | 0.00 (-0.00, 0.00), 0.6251 | 0.00 (-0.00, 0.00), 0.2536 |
| Total cholesterol (mg/dl) | -0.00 (-0.00, -0.00), <0.0001 | -0.00 (-0.00, -0.00), 0.0069 | -0.01 (-0.02, 0.00), 0.2205 |
| LDL-C (mg/dl) | -0.00 (-0.00, -0.00), 0.0083 | -0.00 (-0.00, -0.00), 0.0233 | 0.01 (-0.00, 0.02), 0.2290 |
| Direct HDL-C (mg/dl) | -0.00 (-0.00, -0.00), <0.0001 | -0.00 (-0.00, -0.00), 0.0153 | 0.01 (-0.00, 0.02), 0.2183 |

**Supplementary Table 1 Multivariate linear regression models for total bone density.**

Model 1: no variable adjustment.

Model 2: age, sex, and race were adjusted, but the model did not adjust for the variables themselves.

Model 3: age, sex, race, education, smoking status, diabetes, CKD, PIR, BMI,albumin, ALT, AST, vitamin D, BUN, total calcium, creatinine, serum glucose, glycohemoglobin,phosphorus, uric acid, eGFR, ACR, total cholesterol, triglyceride, LDL-C, and direct HDL-C were adjusted, but the model did not adjust for the variables themselves.
