# Supplementary material for: The relationship between weight-adjusted-waist index and total bone mineral density in adults aged 20-59
Source: Front Endocrinol (Lausanne). 2023 Nov 23;14:1281396. doi: 10.3389/fendo.2023.1281396 (PMC10701523; doi:10.3389/fendo.2023.1281396)
Supplement: Supplementary file 2 [file Table_2.docx]

| **Supplementary Table 2**  **Basic information of subjects per cycle year** | | | | | |
| --- | --- | --- | --- | --- | --- |
|  | **2011-2018** | **2011-2012** | **2013-2014** | **2015-2016** | **2017-2018** |
|  | **N= 10,372** | **N= 2,596** | **N= 2,955** | **N=** **2,681** | **N=** **2,140** |
| Age (year) | 38.98 ± 11.65 | 39.20 ± 11.79 | 38.84 ± 11.48 | 38.87 ± 11.51 | 39.01 ± 11.85 |
| **Sex (%)** |  |  |  |  |  |
| Male | 51.25 | 52.51 | 51.88 | 49.99 | 50.48 |
| Female | 48.75 | 47.49 | 48.12 | 50.01 | 49.52 |
| **Race (%)** |  |  |  |  |  |
| Mexican American | 10.77 | 9.30 | 11.23 | 11.48 | 11.05 |
| Other Hispanic | 7.63 | 6.97 | 6.82 | 8.12 | 8.77 |
| Non-Hispanic White | 60.12 | 63.27 | 61.33 | 58.53 | 56.97 |
| Non-Hispanic Black | 11.56 | 12.08 | 11.25 | 11.91 | 10.98 |
| Other Race | 9.92 | 8.38 | 9.38 | 9.97 | 12.21 |
| **Education level(%)** |  |  |  |  |  |
| Less than high school | 4.08 | 3.84 | 3.91 | 5.48 | 3.02 |
| High school or GED | 31.18 | 30.52 | 32.82 | 29.03 | 32.30 |
| Above high school | 64.73 | 65.64 | 63.24 | 65.48 | 64.67 |
| Unknown | 0.01 | 0.00 | 0.03 | 0.00 | 0.02 |
| **Smoking status(%)** |  |  |  |  |  |
| Never | 59.57 | 57.24 | 59.78 | 60.15 | 61.26 |
| Former | 18.99 | 19.25 | 17.73 | 18.94 | 20.27 |
| Current | 21.40 | 23.44 | 22.49 | 20.85 | 18.46 |
| Unknown | 0.03 | 0.07 | 0.00 | 0.06 | 0.00 |
| **Diabetes (%)** |  |  |  |  |  |
| Yes | 9.26 | 9.03 | 9.53 | 10.29 | 8.05 |
| No | 90.74 | 90.97 | 90.47 | 89.71 | 91.95 |
| **CKD (%)** |  |  |  |  |  |
| Yes | 9.17 | 9.35 | 9.79 | 8.82 | 8.61 |
| No | 90.83 | 90.65 | 90.21 | 91.18 | 91.39 |
| PIR | 2.93 ± 1.67 | 2.86 ± 1.69 | 2.92 ± 1.69 | 2.97 ± 1.65 | 2.98 ± 1.65 |
| Albumin (g/dl) | 4.32 ± 0.34 | 4.36 ± 0.33 | 4.33 ± 0.32 | 4.42 ± 0.33 | 4.15 ± 0.32 |
| ALT (U/L) | 26.07 ± 19.59 | 26.39 ± 19.54 | 26.80 ± 21.97 | 26.77 ± 18.46 | 24.06 ± 17.54 |
| AST (U/L) | 25.17 ± 17.47 | 26.28 ± 17.10 | 25.76 ± 21.18 | 25.82 ± 16.06 | 22.52 ± 13.74 |
| Vitamin D (nmol/L) | 66.19 ± 25.97 | 66.72 ± 26.92 | 64.76 ± 24.83 | 65.98 ± 25.63 | 67.58 ± 26.54 |
| Total calcium (mg/dl) | 9.37 ± 0.35 | 9.38 ± 0.33 | 9.45 ± 0.37 | 9.35 ± 0.33 | 9.29 ± 0.34 |
| Phosphorus (mg/dl) | 3.72 ± 0.56 | 3.74 ± 0.55 | 3.82 ± 0.58 | 3.71 ± 0.55 | 3.60 ± 0.54 |
| Serum glucose (mg/dl) | 97.00 ± 32.14 | 95.91 ± 30.72 | 98.72 ± 35.04 | 96.82 ± 32.83 | 96.34 ± 28.99 |
| Glycohemoglobin (%) | 5.52 ± 0.91 | 5.53 ± 0.90 | 5.48 ± 0.93 | 5.55 ± 0.97 | 5.52 ± 0.83 |
| Uric acid (mg/dl) | 5.33 ± 1.36 | 5.36 ± 1.35 | 5.35 ± 1.36 | 5.33 ± 1.34 | 5.26 ± 1.40 |
| BUN(mg/dl) | 12.76 ± 4.24 | 11.86 ± 4.14 | 12.13 ± 4.07 | 13.58 ± 4.30 | 13.60 ± 4.16 |
| Creatinine (mg/dl) | 0.86 ± 0.29 | 0.86 ± 0.24 | 0.87 ± 0.32 | 0.84 ± 0.31 | 0.85 ± 0.31 |
| ACR (mg/g) | 12.24 ± 26.06 | 12.29 ± 27.16 | 12.63 ± 25.22 | 12.19 ± 25.91 | 11.79 ± 25.95 |
| eGFR (mL/min/1.73 m2) | 94.37 ± 21.28 | 93.71 ± 20.74 | 92.80 ± 20.43 | 96.54 ± 22.38 | 94.58 ± 21.41 |
| Triglyceride (mg/dl) | 119.08 ± 115.77 | 131.54 ± 107.60 | 121.26 ± 146.59 | 111.08 ± 79.12 | 110.71 ± 113.44 |
| Total cholesterol (mg/dl) | 190.86 ± 40.03 | 194.50 ± 40.61 | 189.20 ± 41.88 | 190.86 ± 39.71 | 188.86 ± 37.07 |
| LDL-C (mg/dl) | 113.72 ± 33.99 | 116.34 ± 35.36 | 112.28 ± 33.75 | 113.96 ± 35.00 | 112.26 ± 31.44 |
| Direct HDL-C (mg/dl) | 52.75 ± 15.60 | 51.71 ± 14.60 | 52.31 ± 15.44 | 53.95 ± 17.26 | 53.08 ± 14.81 |
| Weight (kg) | 82.43 ± 20.70 | 82.11 ± 19.69 | 82.63 ± 21.20 | 82.74 ± 20.87 | 82.19 ± 21.00 |
| Waist circumference (cm) | 97.56 ± 16.25 | 97.06 ± 15.52 | 97.64 ± 16.43 | 98.00 ± 16.34 | 97.51 ± 16.70 |
| BMI (kg/m^2^) | 28.78 ± 6.66 | 28.46 ± 6.25 | 28.66 ± 6.68 | 29.06 ± 6.86 | 28.99 ± 6.84 |
| Total BMD (g/cm^2^) | 1.12 ± 0.11 | 1.12 ± 0.10 | 1.12 ± 0.11 | 1.11 ± 0.11 | 1.12 ± 0.10 |
| Weight-adjusted-waist index | 10.79 ± 0.78 | 10.75 ± 0.77 | 10.79 ± 0.76 | 10.82 ± 0.79 | 10.80 ± 0.81 |

The mean ± SD is used to express continuous variables. (%) is used to express categorical variables.

Abbreviations: GED, general educational development; CKD, chronic kidney disease;PIR, ratio of family income to poverty; BMI, body mass index; LDL-C, low-density lipoprotein cholesterol; BMD, bone mineral density; HDL-C, direct high-density lipoprotein cholesterol; AST, aspartate transaminase; ALT, alanine transaminase; Vitamin D, 25OHD2 + 25OHD3;BUN, blood urea nitrogen; ACR, albumin: creatinine ratio; eGFR, estimated-glomerular filtration rate.
